# *Anaplasma ovis* Prevalence Assessment and Cross Validation Using Multiparametric Screening Approach in Sheep from Central Tunisia

**DOI:** 10.3390/pathogens11111358

**Published:** 2022-11-15

**Authors:** Sihem ElHamdi, Moez Mhadhbi, Mourad Ben Said, Amine Mosbah, Mohamed Gharbi, Imen Klabi, Monia Daaloul-Jedidi, Hanène Belkahia, Rachid Selmi, Mohamed Aziz Darghouth, Lilia Messadi

**Affiliations:** 1 Laboratory of Microbiology and Immunology, National School of Veterinary Medicine, IRESA & University of Manouba, Sidi Thabet 2010, Tunisia; 2 Parasitology Laboratory, National School of Veterinary Medicine, IRESA & University of Manouba, Sidi Thabet 2010, Tunisia; 3 Department of Basic Sciences, Higher Institute of Biotechnology of Sidi Thabet, University of Manouba, Sidi Thabet 2010, Tunisia; 4 National School of Veterinary Medicine, IRESA & University of Manouba, Sidi Thabet 2010, Tunisia

**Keywords:** *Anaplasma ovis*, sheep, central Tunisia, infection monitoring, multiparametric screening, cross validation, *msp4* gene analysis

## Abstract

We conducted a 5-month-long screening of *Anaplasma* spp. and *Anaplasma ovis* infection in sheep from central Tunisia. During this longitudinal study, we investigated the infection dynamics using both direct and indirect assessments validated with a polymerase chain reaction (PCR) as the gold standard method. The experimental design included 84 male lambs aged from 6 to 8 months, and 32 ewes, both chosen randomly from June to November with a periodicity of 2 weeks approximately between June and September, and 1 month between September and November. A total of 9 field visits were carried out in this period during which animals were clinically examined and biological samples were extracted. Thus, a total of 716 blood smears, 698 sera from the nine sampling dates, as well as 220 blood samples from the first and the ninth sampling dates were collected from apparently healthy lambs and ewes, respectively, and analyzed by competitive enzyme-linked immunosorbent assay (cELISA) and polymerase chain reaction (PCR) assay, for the detection of *Anaplasma* antibodies and *A. ovis* DNA, respectively. Sera were analyzed by competitive enzyme-linked immunosorbent assay (cELISA) and PCR, for the detection of *Anaplasma* antibodies and *A. ovis* DNA, respectively. The *Anaplasma* spp. initial seroprevalence rate was 33.3% in lambs and 100% in ewes, and it then flowed in an upward trend to reach a maximum of 52.6% in lambs, whereas in ewes, the *Anaplasma* spp. seroprevalence rate remained unchanged and equal to 100%. Meanwhile, the *A. ovis* initial molecular prevalence was 22.6% at the first visit and 26.3% at the last visit in lambs, whereas in ewes, the molecular prevalence rates of *A. ovis* were higher in both the first and the last visit estimated at 100% and 85.7%, respectively. The Kappa coefficient between cELISA and PCR indicated a moderate level of agreement on the first sampling date (0.67) and a low agreement level on the last (0.43). Furthermore, an exploratory data analysis using a multimodal machine learning approach highlighted the underlying pattern of each analytical technique used in this study. In this prospect, we were able to establish the performance of each technique at detecting *Anaplasma* spp. in sheep. The combination of these approaches should improve the field assessment while promoting a data-based decision in precision epidemiology. The genetic follow-up test relevant to *A. ovis msp4* sequences revealed three different genotypes, two of which were previously described in Italy.

## 1. Introduction

Ovine anaplasmosis “*sensu stricto*” is an infectious disease caused by the tick-borne obligatory intraerythrocytic pathogen *Anaplasma ovis (A. ovis)* [1]. This rickettsial bacterium is considered as a highly prevalent tick-borne pathogen of sheep, goats [2], and wild ruminants [3]. *A. ovis* was reported in all continents and has a widespread distribution [4]. It has also extended to North Africa where it became a major livestock health issue magnified by the intensification of the reservoir hots and tick vector population [5]. Infection is usually subclinical and, occasionally, goats may develop rickettsiaemia associated with fever, severe anemia, weight decrease, milk yield drop, and for some animals, abortions or death during acute infections [6]. Interestingly, *A. ovis* infection may pave the way to more severe health issues with life-threatening consequences and financial burden as well [7,8].

Diagnosis is commonly based on a direct *Anaplasma* examination of Giemsa-stained blood smears under a microscope; even if this method is convenient and straightforward, it is only relevant during the early infection phase when *A. ovis* concentration is well above the detection limit of 106 infected erythrocytes per ml [9], and therefore it remains much less sensitive during chronic and/or asymptomatic carriers’ infection status. To counteract this setback a competitive ELISA (cELISA) was proposed. It is based on an antibody binding to the recombinant *Anaplasma* major surface protein 5 (Msp5), which seems to be conserved in all *Anaplasma* species [10]. This technique was used to detect both acute and chronic *A. ovis* infections in goats [11], sheep, and wildlife [12], and both *A. marginale* and *A. centrale* infections in cattle [10]. However, and despite its sensitivity, this serological test may lack specificity according to the spectrum of *Anaplasma* species present in the investigated regions.

More recently, a molecular test based on *msp4* gene amplification has shown higher performances at detecting *Anaplasma* infection. However, PCR-based techniques are not scalable and are yet to be deployable on the field.

In the meantime, the only reasonable alternatives for real-time detection of *Anaplasma* infection on the field remain blood smear screening and cELISA testing. In fact, PCR/cELISA, blood smear/cELISA, and PCR/blood smear combinations after fine-tuning and cross validation could be more appropriate approaches for the investigation of *Anaplasma* infections, especially in a complex epidemiological context [6,13,14].

This cross validation has become a real analytical asset for the real-time detection of critical endemics such as *A. ovis*, especially when prevalence rates are influenced by external and internal factors such as bioclimatic zone, season, age, animal breed, and/or tick burdens [15,16,17,18].

Given the endemicity of *A. ovis* infection in sheep in Tunisia and the variability of the diagnosis and reporting systems, a general robust framework should be implemented nationwide to reduce misinterpretations while keeping track of the infection’s dynamics and its basic epidemiological features.

In the present study, we aimed to investigate the *A. ovis* epidemiology in central Tunisia using a multifactorial screening approach, while testing for the available analytical combination that fits best with the local and regional context.

## 2. Materials and Methods

### 2.1. Investigated Flock and Sheep

**Figure 1:**
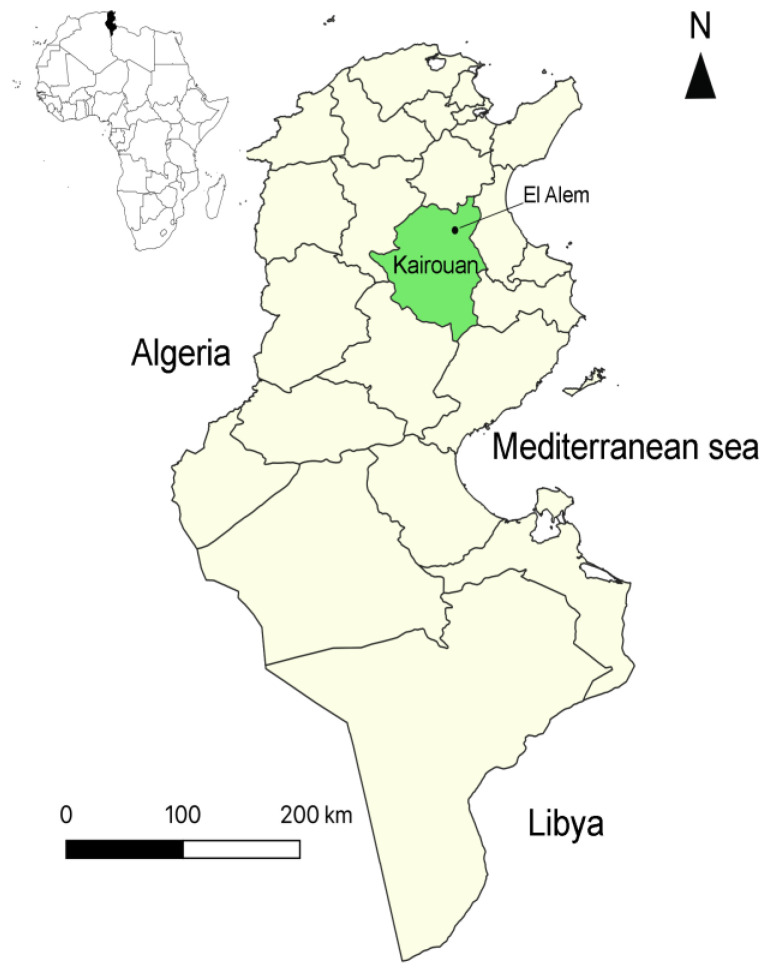
Map showing the Tunisian studied region.

The present study was carried out at the El-Alem agricultural complex located in the Sbikha Delegation of the Kairouan Governorate, central Tunisia. This region is characterized by an arid climate (25 °C of temperature, 150 mm of annual rainfall averages) and a mean altitude of 60 m above sea level (latitude 35°40′ N, longitude 10°06′ E) (Figure 1). The main activity of the El-Alem complex is focused on cattle, sheep, and camel breeding intended for the production of milk and meat. The sheep flock of the El-Alem complex had a previous history of clinical anaplasmosis due to *A. ovis* infection in animals during the first summer season, confirmed by blood smear [15,19]. Cases of anaplasmosis caused by *A. ovis* have also been demonstrated in ewes during previous seasons. Sheep were reared according to a traditional improved semi-intensive system, animals which are of the Barbarin breed were maintained under open shelters, and ewes grazed on natural grasslands. All animals were subjected monthly to anthelmintic and external acaricidal treatment from May to November.

In this study, 84 male fattening lambs at the first summer season and 32 adult ewes were randomly selected for the flock out of a total number of 1041 heads. The lambs were between 6 and 8-months-old and they were kept in a pen fenced with barbed wire and covered with a litter of straw. They did not graze and were fed and watered on site. The ewes were kept in the same conditions but in separate shelters and they had, as already mentioned, access to pasture. All sampled animals were apparently in good health at the start of the study.

### 2.2. Sampling Strategy

Blood and sera samples were collected from the selected animals (84 lambs and 32 ewes) during the summer season (from June to November). Lambs at the first disease season were closely monitored over nine sampling dates. The sampling periodicity was approximately 2 weeks from June to September, and then 30 days from September to November. At each sampling date, a brief clinical examination and weighing were carried out for all lambs. At the same time, a check for the possible presence of ticks was performed at each visit from the surveyed lambs. The ewes were only sampled on the first and the last (ninth) visit, to have a global view of the infection rates. During the period of the study, eight lambs and four ewes were taken out from the survey.

### 2.3. Blood Sampling, Obtaining Sera, and DNA Extraction 

Blood was collected from the jugular vein of each animal on dry and EDTA vacutainer tubes. The sera were extracted (dry tubes) after centrifugation at 3000× *g* rpm for 10 min, and sera were stored at −20 °C until used. EDTA tubes were used to extract the DNA from 300 µL of blood with the Wizard^®^ Genomic DNA purification kit (Promega, Madison, WI, USA) according to the recommendations of the manufacturer. Purified DNA was stored at −20 °C until used.

### 2.4. Blood Smears

Screening for *A. ovis* infection was performed by examining Giemsa-stained blood smears. The observation was made under an optical microscope with the objective × 100 with a drop of immersion oil on about 50 fields.

The identification of *A. ovis* was based on the following morphological characters: infected erythrocytes had been detected by the presence of inclusion bodies located centrally, submarginally, or less frequently in a marginal position [20,21].

### 2.5. Serological Analysis

A total of 698 sera samples were tested using the *Anaplasma* Antibody Test Kit, cELISA v2 (VMRD, Pullman, WA, USA) according to the manufacturer’s instructions. This recombinant major surface protein 5 (rMSP5)-based competitive ELISA (cELISA) was used for the detection of antibodies against *A. marginale*, *A. centrale*, and *A. ovis* MSP5 antigens [22]. It had a sensitivity and a specificity of 100% and 99.7%, respectively, for these three species [23]. The results were presented as percent inhibition (%I) and calculated by [(1 − OD620 of test sample)/(OD620 of negative plate control)] × 100, where OD620 was the optical density at 620 nm. The sample was considered positive if % I ≥ 30.

The cumulative prevalence was calculated by counting the number of lambs infected at least once during the entire follow-up period. 

### 2.6. Specific Molecular Detection of A. ovis

The EHR16SD/EHR16SR primer set was used as a catch-all PCR (PCR screening): it amplified 345 bp of all *Anaplasmataceae* 16S rRNA gene [24] (Table 1). PCR reactions were performed on 220 DNA samples from 32 ewes and 84 lambs for the first sampling date, and 28 ewes and 76 lambs for the last sampling date in a final volume of 50 µL. In particular, PCR tubes contained 0.125 U/µL Taq DNA polymerase (Biobasic Inc., Markham, Canada), 1 × PCR buffer, 1.5 mM MgCl_2_, 0.2 mM dNTPs, 2 µL (50 to 150 ng) genomic DNA, and 0.5 µM of primers. The thermal program profile was as described by Parola et al. (2000) [24].

Positive 16S rRNA samples were used as a positive control of *A. ovis* infections using a single PCR with msp43 and msp45 primers that amplified 852 bp of the *msp4* gene [13] (Table 1). PCR amplifications were performed using a mixture of the following reagents: 1 × Taq MasterMix (Vivantis, Oceanside, CA, USA) (containing 1 × PCR buffer, 1.5 mM MgCl_2_, 0.2 mM dNTPs, and 0.125 U/µL Taq DNA polymerase), 2 µL (50 to 150 ng) DNA, 0.5 µM of the primers, and milliQ sterile water to a total volume of 50 µL. To ensure the accuracy of the method, negative (distilled sterile DNA free water) and positive controls (*A. ovis* PCR amplicons) were used in each PCR run every 10 samples. Thermal cycling reactions were performed in an automated DNA thermal cycler (GeneAmp PCR System 2700, Applied Biosystems, Woodlands, Singapore) using the following conditions: an initial denaturation at 94 °C for 30 s, followed by 35 cycles (a denaturation at 94 °C for 30 s, an annealing step of 30 s at 60 °C, and extension at 68 °C for 1 min), and a final extension of 68 °C for 7 min. Amplicons were electrophoresed in 1.5% agarose gel.

### 2.7. Data Mining

Four analytical test results (blood smear, serology, and both performed PCR) were collected during the 144 days and compiled with weather data and tick presence observations to run a full exploratory data analysis (EDA). The EDA included ordinary least squares (OLS) regression, correlation, and clustering using Pearson coefficient and ward D distance assessment, respectively. All biological tests were cross validated using a customized machine learning pipeline designed and run on Multiverse AI ^®^ cloud computing platform available on (https://multiversai.com/ accessed on 16 October 2022).

Test’s metrics and performances were reported into confusion matrices.

### 2.8. DNA Sequencing and Phylogenetic Analysis

Four selected PCR amplicons, two from the first sampling date (one lamb and one ewe) and two from the ninth sampling date (one lamb and one ewe), were purified with the GF-1 Ambi Clean kit (Vivantis, California, USA) according to the manufacturer’s instructions. Purified amplicons were sequenced in both directions using the same primers as for the *A. ovis msp4*-specific PCR amplifications (Table 1). The reaction was realized with a conventional Big Dye Terminator cycle sequencing ready reaction kit (Perkin Elmer, Applied Biosystems, Foster City, USA) and ABI3730XL automated DNA sequencer by Macrogen Europe (Amsterdam, The Netherlands). The evaluation of chromatograms was performed by using Chromas Lite v 2.01. 

Multiple sequence alignments of the amplicons and nucleotides’ translation were performed using DNAMAN program (Version 5.2.2; Lynnon Biosoft, Quebec, Canada). A BLAST analysis was performed in GenBank to compare the published sequences with ours (http://blast.ncbi.nlm.nih.gov/, accessed on 24 June 2022) [25]. The DNAMAN program was used to build a phylogenetic tree based on the distance method using the neighbor-joining (NJ) algorithm of Saitou and Nei (1987) [26] with a bootstrap of 1000 iterations. Four *msp4* amplicons were submitted to GenBank under accession numbers from MZ073666 to MZ073669.

### 2.9. Statistical Analysis

For each prevalence rate, a 95% exact confidence interval (CI) was calculated.

The regression test according to time (R^2^) was performed to evaluate the seroprevalence throughout the experimental study.

The χ^2^ test with a 5% threshold was used to compare prevalence rates estimated by the different diagnosis techniques during visits.

The level of agreement between the four used techniques was estimated with a Kappa test. 

With regard to data mining, all statistical assessments were designed and run on the Multiverse ai ^®^ cloud computing platform (https://multiversai.com/ accessed on 16 October 2022).

## 3. Results

### 3.1. Blood Smears

**Figure 2:**
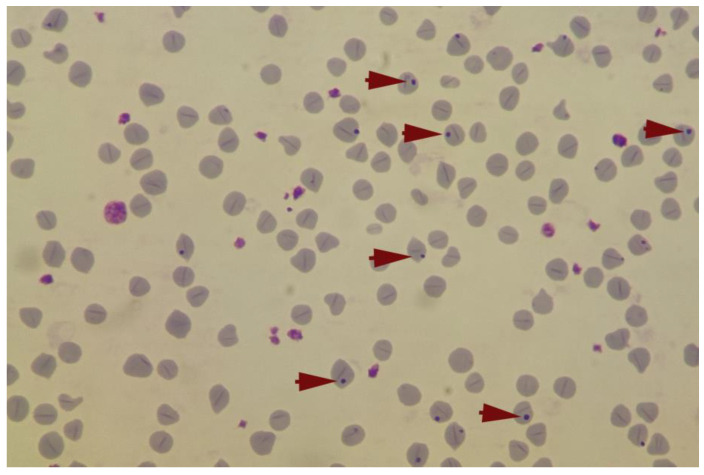
*A. ovis* in infected erythrocytes from lambs’ blood smear, Giemsa staining.

The parasitological diagnosis identified 34 infected animals throughout the follow-up (Figure 2), i.e., a cumulative overall prevalence of infection of 40.48%.

**Figure 3:**
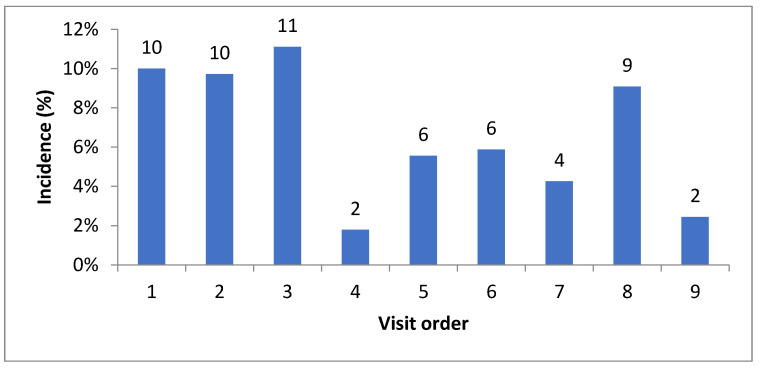
Incidence of *A. ovis* infection per visit.

The prevalence of infection was maximum from the second half of July until the end of August, and its variation was not statistically significant (*p* = 0.058). We estimated the incidence of new cases of *A. ovis* infection confirmed at each visit and it varied from 2 to 11% (Figure 3).

### 3.2. Seroprevalence of Anaplasma spp. Antibodies in Sheep

**Figure 4:**
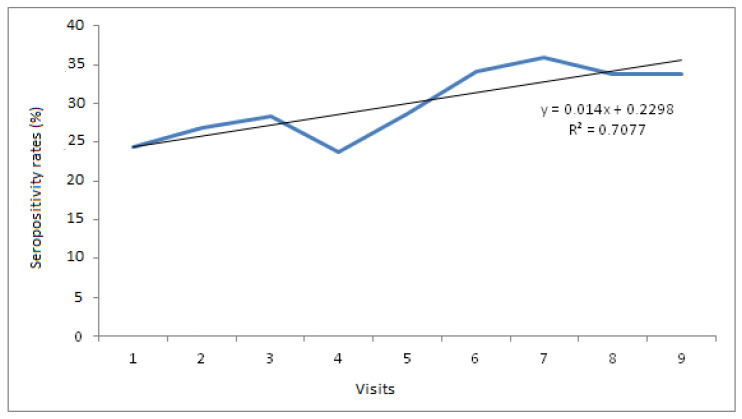
Regression test applied to the serological results of lambs obtained during the nine sampling dates.

In lambs, the *Anaplasma* spp. seropositivity varied according to sampling dates; it began with a globally moderate seroprevalence (33.3%) at the first sampling date in June, then it increased gradually from one sampling date to another and reached its maximum during the last sampling date (52.6%). By contrast, in ewes, all the animals were positive at the first visit and remained so at the last sampling date (Table 2). Referring to the regression test according to time, the seroprevalence increase was statistically significant in lambs (R^2^ = 0.7077) (Figure 4). On the other hand, the cumulative seroprevalence in lambs was equal to 62.02%.

### 3.3. Molecular Prevalence of Anaplasmataceae and A. ovis in Sheep

By using the PCR assay, a total of 24 out of 84 (28.6%) lambs sampled during the first sampling date were found to be infected with *Anaplasmataceae*, while all the ewes tested positive for these bacteria (32/32). At the ninth sampling date, the *Anaplasmataceae* prevalence rate increased in lambs and reached 36.8% (28/76). However, this increase was not statistically significant (χ^2^ = 1.244 < 3.84; *p* > 0.05). In the case of ewes, this prevalence rate was similar to that estimated during the first sampling date (28/28, 100%) (Table 3).

The *A. ovis*-specific PCR revealed at the first sampling date an infection rate of 22.6% (19/84 lambs), which was slightly below the positivity rate for the *Anaplasmataceae* PCR. This rate increased at the ninth sampling date to 26.3% (20/76); however, this increase was not statistically significant (χ^2^ = 0.295 < 3.84; *p* > 0.05). In ewes, infection with *A. ovis* showed a maximum prevalence rate at the first sampling date (100%, 32/32 ewes). At the last sampling date, although showing a statistically significant decrease (χ^2^ = 4.897 > 3.84; *p* < 0.05), this prevalence rate remained high (24/28 ewes, 85.7%) (Table 3).

Eight lambs were lost during the follow-up period (slaughtered or dead), and only one lamb was positive in the *A. ovis* PCR and serology; furthermore, another lamb was slaughtered after developing clinical signs of cerebral coenurosis. Accordingly, we could deduce that the death of these lambs was not caused by anaplasmosis.

### 3.4. Comparison of Serological, Molecular, and Blood Smear Methods in Lambs and Exploratory Data Analysis

A comparison of the *Anaplasma* spp. PCR results with those of serology (Table 3) revealed that almost all samples positive at the PCR were also positive for cELISA. Nevertheless, nine and fifteen samples that were negative at PCR amplification were positive in cELISA for the first and ninth visit, respectively. The level of agreement between the two techniques was moderate (0.67) to weak (0.44) for the first and last sampling dates, respectively (Table 3). Very similar levels of agreement were also obtained between serology and *A. ovis*-specific PCR (Table 4).

Likewise, the agreement level of the results of blood smear and *A. ovis* PCR was weak in the first and the ninth visit, namely 0.53 and 0.43, respectively (Table 5), and the agreement level of the results of the blood smear and serology was lower in the first and the ninth visit (0.32 and 0.24, respectively) (Table 6).

**Figure 5:**
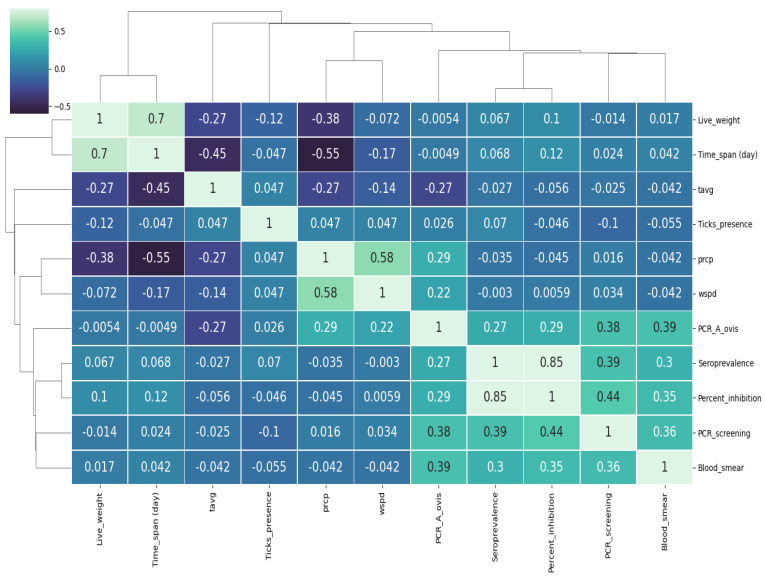
Heatmap of multiparametric correlation to assess *A. ovis* infection in lambs. Time-span: duration; tavg: average temperature; prcp: precipitation; wspd: wind speed; percent_inhibition: the inhibition percent of cELISA technique; PCR screening: the catch-all PCR.

On the other hand, OLS regression and correlations tests revealed a high match between serological and PCR tests for both genus and species assessments (R^2^ = 0.8) (Figure 5). 

**Figure 6:**
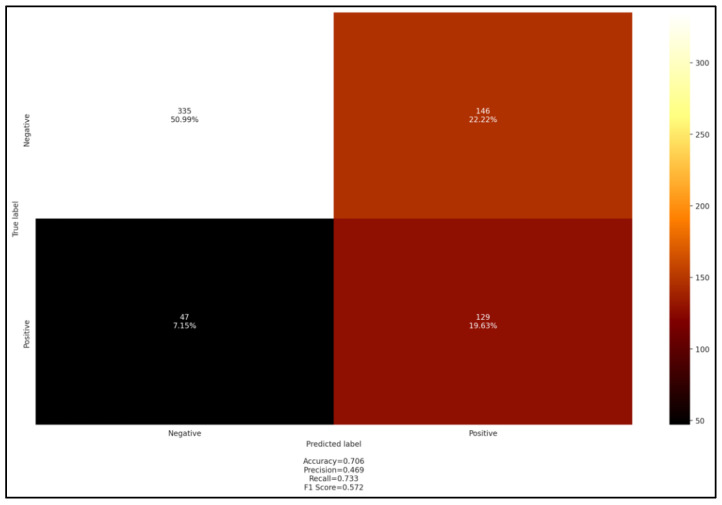
Confusion matrix cELISA *versus* PCR screening.

The PCR screening had the highest coefficient in the OLS regression test compared to other parameters. These observations were confirmed by the hierarchical clustering test (Figure 5), which highlighted a relative proximity between the 4 analytical tests (blood smear, serology, and both performed PCR), whereas tick presence at the site of infection belonged to the climatic data cluster. The prevalence of *Anaplasma* followed an upward trend with a maximum seroprevalence of 52.6% as well as molecular and hematological tests. The serological test showed high performance in detecting *Anaplasma* spp., with both accuracy and recall of 0.7, whereas other metrics were relatively low, namely, precision and F1 score, which were both close to 0.5 (Figure 6).

**Figure 7:**
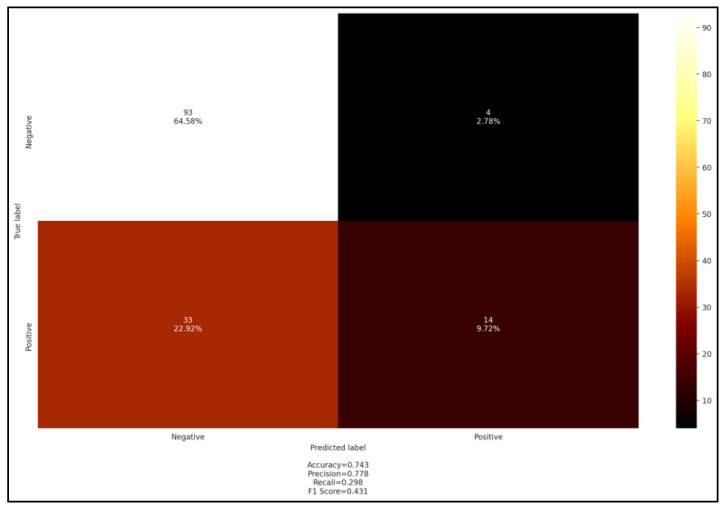
Confusion matrix blood smear *versus* PCR screening.

These performances were partly reversed for the blood smear test where both accuracy and precision exceeded 0.74; however, the recall and F1 score were below 0.5 (Figure 7).

**Figure 8:**
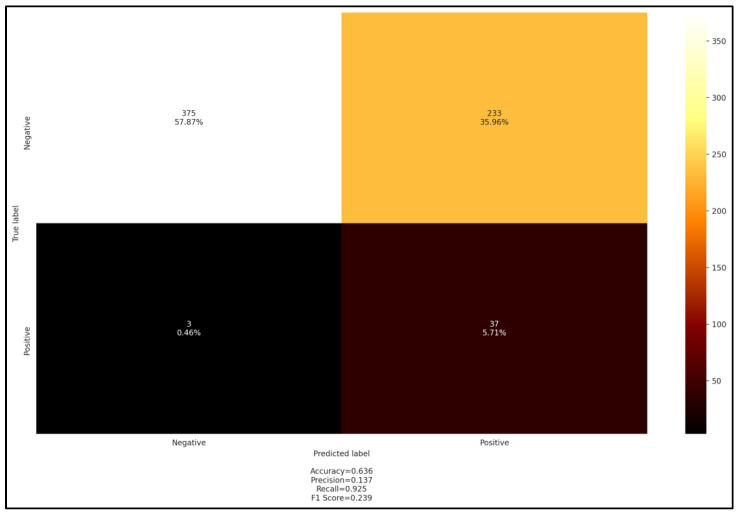
Confusion matrix cELISA versus PCR *A. ovis*.

Otherwise, the comparison of serological test results with *A. ovis* PCR results revealed a high recall score of 0.9, while all other metrics were low and below the cutoff of 0.5 (Figure 8).

### 3.5. Genetic Diversity Analysis and Phylogenetic Study of A. ovis Isolates

*Anaplasma ovis* infection was confirmed by partial sequencing of the *msp4* gene from the randomly selected four positive samples. Alignment of these sequences revealed three different genotypes with three distinctions of nucleotide positions (GenBank accession numbers MZ073666 to MZ073669). Each nucleotide change conferred an amino acid variation (Table 7). The AoGAlam1 genotype (MZ073666 and MZ073667) represented by Lamb15v1 and Lamb28v9 isolates revealed 100% homology with the genotype AOG2 represented by “Italy147” *A. ovis* strain (AY702924) (Table 7). The AoGAlam2 genotype (MZ073668) represented by the Ewe12v1 isolate showed 100% sequence identity to the genotype AOG3 represented by “Italy20” *A. ovis* strain from Sicilian sheep (AY702923) (Table 7). The third genotype AoGAlam3 represented by the Ewe20v9 isolate (GenBank accession number MZ073669) was 99% identical to AoGOv1 and AoGGo1 genotypes (GenBank accession numbers KM285218 and KM285217, respectively) from different *A. ovis* isolates infecting sheep and goats from the north of Tunisia, and to the genotype AOG3 represented by “Italy20” from Italian sheep (AY702923) (Table 7).

**Figure 9:**
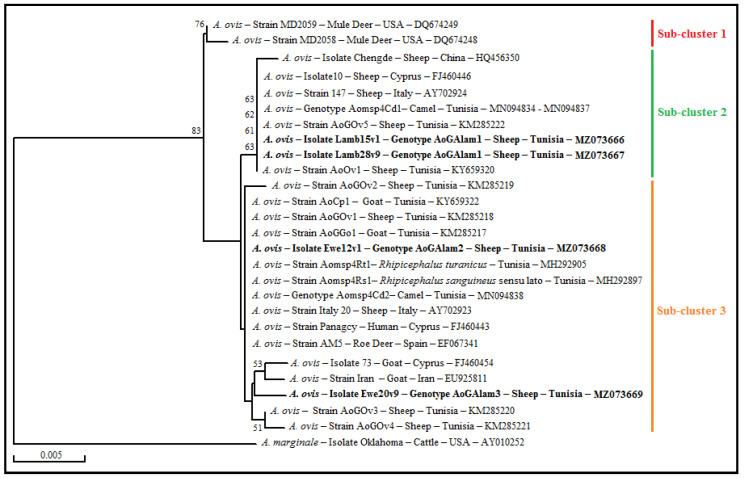
Neighbor-joining tree based on the alignment of partial *msp4* sequences (719 bp) of *Anaplasma ovis*. Multiple sequence alignments were generated with DNAMAN program (Version 5.2.2; Lynnon Biosoft, Quebec, Canada). Numbers associated with nodes represent the percentage of 1000 bootstrap iterations supporting the nodes (only percentages greater than 50% were represented). *A. ovis* sequences obtained in the present study are highlighted in bold. The host or vector, the strain or isolate name, the country of origin and the GenBank accession number are indicated.

A phylogenetic tree was performed on the basis of the alignment of msp4 partial sequences of the four isolates revealed in this study with those from available *A. ovis* strains and isolates found in GenBank (Figure 9). All *A. ovis* strains and isolates clustered in a single *A. ovis* group formed by three sub-clusters, relatively distant from the *A. marginale* reference strain (AY010252) added as an out-group. Our isolates were clustered into two sub-clusters (Figure 9). Isolates Lamb15v1 and Lamb28v9 were found in sub-cluster 2 with those from sheep located in Mediterranean countries such as Italy, Cyprus, and Tunisia and with four isolates (MN094834-MNN094837), represented by genotype Aomsp4Cd1, infecting Tunisian camels. The isolate Ewe12v1 clustered in the third sub-cluster with the *A. ovis* Panagcy strain found in a human from Cyprus (FJ460443), and with other isolates infecting small ruminants and their associated *Rhipicephalus* ticks and camels from Tunisia and/or neighboring countries such as Italy and Spain. The isolate Ewe20v9 also clustered in the third cluster with other strains infecting small ruminants from the north of Tunisia, Iran, and Cyprus (Figure 9).

## 4. Discussion

We selected a flock where clinical anaplasmosis was already documented in fattening and replacement lambs in the summer season with a peak of cases occurring in late June and early July [19]. For this reason, we focused our investigation on lambs at the beginning of anaplasmosis season and adopted a follow-up period adapted to the period of clinical anaplasmosis cases’ occurrence in the investigated flock as reported by the previous authors.

The competitive ELISA serological test was chosen in this study to follow the occurrence and the persistence of the humoral immune response after *Anaplasma* infection [11]. Serological monitoring according to time offers the opportunity to follow the dynamics of the humoral immune response of animals, especially during their first months. The high seroprevalence noted in lambs points out the frequency of asymptomatic infection and its evolution over time. On the other hand, this serological test does not only allow the estimation of the exposure to *A. ovis* infection but also reveals the exposure to infections with other small ruminant-related *Anaplasma* species [11], such as in particular *Anaplasma bovis* and *A. phagocytophilum*-related species [16,29]. 

For lambs, the seroprevalence increased from 33.3% at the first sampling visit to 52.6% at the ninth sampling visit, and PCR-specific *Anaplasma* spp. revealed a prevalence rate of 28.6% at the first sampling date versus 36.8% at the ninth sampling date. Taken globally, these rates are close to those reported by Torina et al. (2010) [30] in Italy (Sicily) (37%) and Spitalska et al. (2007) in Cyprus (29%) [31]. However, they are significantly higher than those found in Sicily, Italy by Torina et al. (2008) (7%) [32].

These results also reveal a gradual and slow infection of the animals over time. Furthermore, the high prevalence observed at the first sampling date in June clearly indicates that the anaplasmosis transmission season started before the initiation of the survey, presumably since the spring and the beginning of summer, which correspond to the main activity period of *Rhipicephalus sanguineus* sensu lato ticks in central Tunisia [33]. However, lambs monitored in this work are kept permanently parked, making then the risks of animal exposure to ticks low as revealed by almost the lack of detection of any tick specimen during this study on lambs (only two ticks were found on two lambs in the first visit). The discrepancy noted between the high prevalence recorded in PCR and cELISA and the scarcity of detection of tick infestation in lambs means that the use of ixodicide treatments did not prevent anaplasmosis infection, and may indicate a more important epidemiological role of *A. ovis* mechanical transmission via other diptera vectors such as horseflies, stomoxes, and mosquitoes [3,34,35], and possibly also by serial drug injection equipment. This type of transmission, which results in a less massive inoculation of bacteria, might explain the absence of clinical infections in lambs during our survey, as indeed not one of the serologically or PCR-positive lambs suffered from clinical anaplasmosis. 

Interestingly, we have recorded high prevalence rates of almost 100% in *A. ovis* PCR and *Anaplasma* c.ELISA in ewes. Likewise, other authors have found similar results in ewes, such as Ben Said et al. (2015) in northern Tunisia (93.8%) [17], Torina et al. (2010) in Sicily (Italy) (98%) [30], and Hornok et al. (2007) in northern Hungary (99.4%) [6]. Furthermore, Ben Said et al. (2015) [17] reported that sheep exposure to vector ticks on rangelands represents a significant risk factor for *A. ovis* infection. Accordingly, the exposure, in our survey, of ewes to ticks and subsequently to reinfection at each new summer might explain the high prevalence recorded in the present work in this sheep category. Taken altogether, the above results recorded from ewes and lambs support the relevance of further investigations on the relationship between the intensity of infection dynamics and the type of dominant vectors responsible for *A. ovis* transmission. 

On a practical side, the dramatically high prevalence rates recorded in ewes (100% and 85.7% at the first and last visit, respectively) in the present study, despite the application of periodic acaricide treatments, point out the ineffectiveness of the acaricide program to reduce the transmission dynamics of *A. ovis*.

In lambs, we noted a statistically significant but nevertheless low increase in the rate of cumulative seroprevalence along the 6 months of the study. Interestingly, this increase fits into the clinical anaplasmosis season as reported on the same farm by Gharbi et al. (2015). The high seroprevalence of 100% observed in ewes supports the occurrence in the surveyed flock of a state of endemic stability for *A. ovis*. However, the relatively limited prevalence values noted in lambs (26.3%) is not compatible with a typical endemic state since lambs would have been expected to have high serological and infection prevalence levels similar to ewes after the completion of the *A. ovis* transmission season. Furthermore, the moderate serological and infection prevalence recorded in our survey indicates that a high proportion of lambs are neither infected nor immunized against *A. ovis* at the end of their first anaplasmosis season. Accordingly, we would have expected the occurrence of anaplasmosis clinical cases in animals in the second disease season, especially when they are more exposed to ticks at grazing. However, as previously reported by Gharbi et al. (2015) in the same farm, clinical cases of anaplasmosis were diagnosed exclusively in lambs in the first disease season [19]. Taken altogether, these results indicate the occurrence of potential disease risk factors specific to lambs at first summer that are probably playing an important role in disease expression. It is important to investigate these risk factors as they could be of key relevance for developing rational prevention programs. 

The molecular *A. ovis* prevalence dynamics in ewes decreased significantly from 100% to 87.5%, possibly due to reduced *A. ovis* loads outside the transmission season [13]. Accordingly, the peak of transmission might have occurred in ewes before the start of the survey, while lambs seemed to be still exposed to new infections as indicated by the moderate increase in serological and molecular prevalence. These contrasting results between lambs and ewes are possibly indicating the presence of different epidemiological determinants related to the type of dominant vectors, justifying therefore the relevance of further investigations on this aspect of *A. ovis* epidemiology.

In this study, despite high agreement between PCR and cELISA results, two samples were seropositive at cELISA and negative at PCR; indeed, antibodies persist in an animal’s blood for a longer period even with a low bacteraemic level or even after bacterial clearance [36]. This result may also relate to cross reaction with infection by other *Anaplasma* species than *A. ovis* such as *A. marginale* and *A. centrale* [6,28], *A. phagocytophilum* and genetically related strains [29,37], *A. bovis* [16,17], and lastly *A. platys*-like strains [38], which were all identified in sheep in Tunisia. The infection by these species might explain also why 1 out of 5 and 3 out of 8 lambs that were PCR-positive for *Anaplasmataceae* and PCR-negative for *A. ovis* were seropositive at the beginning and the end of the study, respectively. Therefore, the interaction of the above species with *A. ovis* needs to be investigated, particularly in weaned lambs exposed to the risks of clinical anaplasmosis. 

In addition, genetic typing performed in this study showed that although *A. ovis msp4* genotypes may be more conserved than *A. marginale* strains [15,16], genetic diversity occurs in this locus. Indeed, three different genotypes were revealed in four sequenced isolates. This is in agreement with the observations of Ben Said et al. (2015a) in northern Tunisia, Hornok et al. (2007) in Hungary, and Torina et al. (2010) in Italy, which showed a high polymorphism, represented by 5, 5, and 17 genotypes, respectively [6,17,30]. In addition, the predominant AoGAlam1 and AoGAlam2 genotypes, previously described in Italy (AOG2 and AOG3), were detected in sheep from the studied region [13]. 

The comparison of our *msp4* sequences from sheep located in central Tunisia with those from the north previously reported by Ben Said et al. (2015) suggests that the geographic location has no impact on the genetic diversity of the *A. ovis msp4* gene [17]. The presence of identical *A. ovis* sequences in both sites could be explained by animal movements across the country. Similar to the findings of Hornok et al. (2007), the sequence analysis shows nucleotide diversity between sheep of the same location, suggesting that *A. ovis msp4* genotypes may vary between animals of the same geographic location [6].

The revealed isolates can be classified into two different sub-clusters, suggesting multiple introductions of genetically different isolates of *A. ovis* in this studied region. The genotype AoGAlam2 is classified in the third sub-cluster with the *A. ovis* zoonotic strain (Panagcy, FJ460443) which infected a woman in Cyprus [39]. However, no hypothesis can be made at this level, and further studies are recommended to assess the zoonotic potential of this variant in our country.

## 5. Conclusions

In summary, we conclude that ovine anaplasmosis is endemic in the surveyed flock, and probably in other similar husbandries and epidemiological contexts in central Tunisia. The investigated flock is showing the features of an incomplete and fragile enzootic stability state for ovine anaplasmosis. This situation must be considered when planning the management and control programs for this disease in Tunisia. Our work emphasizes the requirement for further studies to investigate the vectors and the wildlife reservoirs for *A. ovis*, the risk factors for disease expression associated with the occurrence of *A. ovis* clinical cases occurring in weaned lambs during the summer as observed in the surveyed flock, the infection by other *Anaplasma* species described in small ruminants and their interaction with *A. ovis*, and finally the economic impacts of these bacterial pathogens in Tunisia. Combining blood smear and serology would avoid the weakness of each test, while synergizing their corresponding strength.

## Figures and Tables

**Table 1:** Primers used in the present study for *A. ovis* detection and the characterization in studied sheep.

Assay	Target Gene	Primer	Sequence (5′-3′)	At (°C)	Amplicon Size (bp)	Reference
Single PCR Anaplasmataceae	16S rRNA	EHR16SF EHR16SR	GGTACCYACAGAAGAAGTCC TAGCACTCATCGTTTACAGC	62	374	[24]
Single PCR *A. ovis*	*msp4*	MSP45 MSP43	GGGAGCTCCTATGAATTACAGAGAATTGTTTAC CCGGATCCTTAGCTGAACAGGAATCTTGC	68	852	[13]

At: annealing temperature.

**Table 2:** Monitoring of Anaplasmataceae spp., *Anaplasma* spp., and *A. ovis* in studied lambs and ewes.

Assay	Target Bacteria	Target	Number and Date of Visit (Positive/Total, Infection Rate, % ± C.I. ^1^)									*p* Value
		Host	1 (20-06)	2 (03-07)	3 (18-07)	4 (31-07)	5 (15-08)	6 (29-08)	7 (12-09)	8 (10-10)	9 (14-11)	(χ^2^)
Blood smear	*A. ovis*	Lambs	9/84 (10.7 ± 0.06)	16/82 (19.5 ± 0.08)	24/82 (29.3 ± 0.09)	23/81 (28.4 ± 0.09)	23/80 (28.7 ± 0.09)	20/78 (25.6 ± 0.09)	18/77 (23.4 ± 0.09)	17/76 (22.4 ± 0.09)	12/76 (15.8 ± 0.08)	0.058 (15.04)
cELISA	*Anaplasma* spp.	Lambs	28/84 (33.3 ± 0.09)	29/83 (35 ± 0.10)	33/83 (36.1 ± 0.10)	30/79 (38 ± 0.10)	36/81 (44.4 ± 0.10)	35/80 (43.8 ± 0.10)	34/79 (43%0.10)	33/76 (43.4 ± 0.11)	40/76 (52.6 ± 0.11)	0.443 (7.89)
		Ewes	32/32 (100%)	−	−	−	−	−	−	−	28/28 (100%)	−
PCR	*Anaplasmataceae* spp.	Lambs	24/84 (28.6% ± 0.09)	−	−	−	−	−	−	−	28/76, (36.8% ± 0.10)	0.266 (1.24)
		Ewes	32/32 (100%)	−	−	−	−	−	−	−	24/28, (85.7% ± 0.10)	0.028* (4.82)
PCR	*A. ovis*	Lambs	19/84, (22.6% ± 0.09)	−	−	−	−	−	−	−	20/76, (26.3% ± 0.09)	0.587 (0.29)
		Ewes	32/32, (100%)	−	−	−	−	−	−	−	24/28, (85.7% ± 0.12)	0.028 * (4.82)

^1^: C.I.: 95% confidence interval. *: statistically significant test. −: not concerned.

**Table 3:** Summary of the molecular and serological detection of *A. ovis* using PCR and cELISA assays in sheep sampled in the center of Tunisia.

Visit	PCR *Anaplasma* spp. ^a^	*Serology* ^b^		Total	Kappa Value	% Agreement ^c^
		Positive	Negative			
1	Positive	21	3	24	0.67	Moderate
	Negative	9	51	60		
	Total	30	54	84		
9	Positive	22	6	28	0.44	Weak
	Negative	15	33	48		
	Total	37	39	76		

^a^ The frequency of positive and negative samples as result of cELISA. ^b^ The frequency of positive and negative samples as result of PCR cross tabulated with cELISA. ^c^ Concordance between *Anaplasma* spp. PCR and cELISA results.

**Table 4:** Summary of detection of *A. ovis* using *A. ovis* PCR and cELISA assays in sheep sampled in the center of Tunisia.

Visit’s Number	cELISA ^a^	*A. ovis* PCR ^b^		Total	Kappa Value [27]	% Agreement ^c^
		Positive	Negative			
1	Positive	21	09	30	0.67	
	Negative	03	51	54		Moderate
	Total	24	65	84		
9	Positive	19	15	34	0.43	
	Negative	06	36	42		Weak
	Total	25	51	76		

^a^ The frequency of positive and negative samples as result of cELISA. ^b^ The frequency of positive and negative samples as result of PCR cross tabulated with cELISA. ^c^ Concordance between *A. ovis* PCR and cELISA results.

**Table 5:** Summary of *A. ovis* detection of using blood smear and PCR in sheep sampled in the center of Tunisia.

Visit’s Number	Blood Smear ^a^	*A. ovis* PCR ^b^		Total	Kappa Value [27]	% Agreement ^c^
		Positive	Negative			
1	Positive	8	0	8	0.53	Weak
	Negative	11	65	76		
	Total	19	65	84		
9	Positive	9	04	13	0.43	Weak
	Negative	11	52	63		
	Total	20	56	76		

^a^ The frequency of positive and negative samples as result of blood smear. ^b^ The frequency of positive and negative samples as result of blood smear cross tabulated with *A. ovis* PCR. ^c^ Concordance between blood smear and *A. ovis* PCR results.

**Table 6:** Summary of *A. ovis* detection of using blood smear and cELISA in sheep sampled in the center of Tunisia.

Visit’s Number	Blood Smear ^a^	*cELISA* ^b^		Total	Kappa Value [27]	% Agreement ^c^
		Positive	Negative			
1	Positive	8	0	8	0.32	Minimal
	Negative	22	54	76		
	Total	30	54	84		
9	Positive	11	2	13	0.24	Minimal
	Negative	27	36	63		
	Total	38	38	76		

^a^ The frequency of positive and negative samples as result of blood smear. ^b^ The frequency of positive and negative samples as result of blood smear cross tabulated with *cELISA*. ^c^ Concordance between blood smear and *cELISA* results.

**Table 7:** Nucleotide and amino acid differences among *msp4* partial sequences from *A. ovis* isolates and strains.

Host	Strain or Isolate	Genotype	Country	GenBank ^1^	*msp4* Nucleotidic Positions (Amino Acid Positions) ^2^					Reference
					230 (77)	244 (83)	470 (157)	476 (159)	532 (178)	
Sheep	Italy 147	AOG2	Italy	AY702924	G (R)	A (S)	C (A)	C	C (L)	[28]
	Italy 20	AOG3	Italy	AY702923	*	*	T (V)	*	*	[28]
	Kh1; Kh2	AoGOv1	Tunisia	KM285218	*	*	T (V)	*	*	[17]
	Al1	AoGOv2	Tunisia	KM285219	G (R)	G (G)	T (V)	*	*	[17]
	Al2	AoGOv3	Tunisia	KM285220	T (I)	*	T (V)	A	*	[17]
	Al3	AoGOv4	Tunisia	KM285221	G (R)	*	T (V)	A	*	[17]
	Kh3	AoGOv5	Tunisia	KM285222	*	*	*	*	*	[17]
Goat	Al1-Al5	AoGGo1	Tunisia	KM285217	*	*	T (V)	*	*	[17]
Sheep	Lamb15v1	AoGAlam1	Tunisia	MZ073666	*	*	*	*	*	Present study
	Lamb28v9	AoGAlam1	Tunisia	MZ073667	*	*	*	*	*	Present study
	Ewe12v1	AoGAlam2	Tunisia	MZ073668	*	*	T (V)	*	*	Present study
	Ewe20v9	AoGAlam3	Tunisia	MZ073669	T (I)	*	T (V)	*	A (I)	Present study

^1^: GenBank accession number; ^2^: numbers represent the nucleotide position starting at translation initiation codon Adenine. Notes: Conserved nucleotide positions with respect to the Italy 147 strain, Sicily (Italy) are indicated with asterisks * (de la Fuente et al., 2005b). Amino acid changes are indicated between parentheses with single letter code. Amino acids: R, Arginine; I, Isoleucine; S, Serine; G, Glycine; V, Valine; A, Alanine; L, Leucine; Nucleotides: T, Thymine; C, Cytosine; G, Guanine; A, Adenine.

## Data Availability

The data that support the findings of this study are available from the corresponding author upon request. The provided GenBank accession numbers for our nucleotide sequences are MZ073666 to MZ073669.
